# Orbital Coupling and Spin Textures of Fe/Pd Thin Films Grown on Si Substrate with High Magnetic Fields

**DOI:** 10.1002/advs.202417810

**Published:** 2025-04-26

**Authors:** Xuefeng Wu, Bin Gong, Wenyu Kang, Li Chen, Xu Li, Yaping Wu, Junyong Kang

**Affiliations:** ^1^ College of Physical Science and Technology Engineering Research Center for Micro‐Nano Optoelectronic Materials and Devices at Education Ministry Key Laboratory of Semiconductors and Applications of Fujian Province Tan Kah Kee Innovation Laboratory (FDIX) Xiamen University Xiamen 361005 China; ^2^ School of Microelectronics Fudan University Shanghai 200433 China; ^3^ Shanghai Integrated Circuit Manufacturing Innovation Center Shanghai 201210 China; ^4^ Ningbo Institute of Materials Technology and Engineering Chinese Academy of Sciences Ningbo 315201 China

**Keywords:** Fe/Pd thin films, High magnetic field, Orbital coupling, Spin polarization, Spin textures

## Abstract

The crystallization and magnetic properties strongly depend on the orbital coupling and spin polarization of magnetic materials. Here, the enhancement and freeze of coupling and polarization of atomic orbitals achieved by high magnetic fields are delineated through the first‐principles calculations. Thus a growth method (under high magnetic fields) is proposed to design the crystallization and magnetic structure of the Fe/Pd thin films. The dynamic processes of film growth are revealed based on the observation of transmission electron microscopy. As the magnetic field increased, the Fe film is found to develop from a unique droplet shape to a 2D growth mode, which is caused by the competition between demagnetization energy and interfacial energy. Furthermore, the improvements achieved by high magnetic fields in macroscopic magnetic properties and ordered magnetic domains are demonstrated, confirming the effective regulation of the orbital coupling and spin textures from this designed method. This work provides a new perspective for understanding the interaction between atomic‐orbital and external magnetic fields and offers a strategy for the preparation of high‐performance magnetoelectric and spintronic devices.

## Graphical abstract

High magnetic fields (HMFs) regulate orbital coupling and spin polarization in Fe/Pd thin films, promoting crystallization and ordered spin textures. HMF‐assisted growth transitions Fe from amorphous to dense 2D structures, enhancing saturation magnetization and domain uniformity. This strategy advances the design of high‐performance spintronic devices via precise magnetic and structural control.

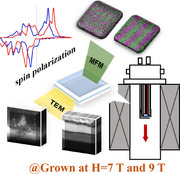

## 1 Introduction

Magnetic thin films have garnered significant attention due to the miniaturization trend in electronic devices and the emergence of spintronics. The construction of electron/spintronic devices usually requires the integration of multiple thin film materials. For instance, spin valves often feature a ferromagnet (FM)/metal (M) structure,^[^ ^1^, ^2^ ^]^ spin‐orbit torque devices utilize FM/non‐magnetic layers,^[^ ^3^, ^4^ ^]^ and magnetic tunnel junctions employ FM/non‐magnetic insulator (I)/FM configurations.^[^ ^5^, ^6^ ^]^ The performance of these devices is significantly influenced by the properties of each thin film and the interfaces between them, especially, the crystal and magnetic structures of the FM layer are critical. Consequently, many efforts have been made to regulate FM films. For example, Kumar et al. examined the effects of annealing on the structural and magnetic properties of Fe thin films sandwiched between Au and Si. They found that besides improvement in the magnetic properties, the annealing also induced a mixing phase at the Fe/Si interface.^[^ ^7^ ^]^ Alternatively, increasing the growth temperature during direct‐current magnetron sputtering was shown to improve the crystallinity of magnetic Fe thin films.^[^ ^8^ ^]^ However, this approach may introduce lattice mismatch at the interface due to the thermal expansion effect and self‐doping diffusion of substrate impurities into the epitaxial layer. Therefore, there remains a pressing need for an efficient and convenient preparation method to produce multilayer films with high‐quality FM films, sharp interfaces, and expected magnetic characters.

The crystal and magnetic properties are essentially determined by the orbital coupling and spin polarization of magnetic materials. This provides a promising idea for regulating FM films. Applying a high magnetic field (HMF) during material growth is a noninvasive and pivotal approach that could effectively interact with the magnetic dipole of FM atoms.^[^ ^1^, ^9^ ^]^ By introducing this magnetic coupling effect, HMF is expected to modulate the thermodynamic and kinetic behaviors of material growth, and thus, determine the crystal orientations,^[^ ^10^, ^11^, ^12^ ^]^ grain sizes,^[^ ^13^ ^]^ the magnetic phase, and spin textures of the films.^[^ ^14^, ^15^ ^]^ Molecular beam epitaxy (MBE) stands as an optimized technique to investigate the impact of HMF on the growth of FM materials and their heterostructures. It overcomes the limitations of solution growth methods that lack precise multilayer control and avoids the susceptible interlayer mixing during the magnetic field annealing. Prior research by Du et al. explored the effects of a magnetic field of 6T on the microstructure and magnetic properties of Fe films deposited via thermal evaporation, revealing enhanced grain size and soft magnetic characteristics.^[^ ^16^ ^]^ However, there is a scarcity of reports on the impact of even stronger magnetic fields on ferromagnetic materials and multilayer film interfaces, and the growth mechanisms under (HMF) remain enigmatic.

In this study, we first predicted the modulation of the growth magnetic field on the orbital coupling, crystallization, and magnetic properties of the Fe films. Then, the HMF‐assisted MBE was used to deposit Fe/Pd films on native oxide Si (100) substrates, with the Fe thin film grown under HMF up to 9T. The morphology and crystalline structures of Fe/Pd films were systematically investigated by transmission electron microscopy to reveal the dynamic process of crystal growth under the HMF. Accordingly, the evolution of growth mode was analyzed through the competition between demagnetization energy and interfacial energy. Additionally, the macroscopic magnetic properties and micro spin texture of the Fe/Pd films were studied, to delve into the underlying mechanisms governing the HMF modulation.

## 2 Results and Analysis

### 2.1 Theoretical Prediction of the Effect of Growth HMF on Fe Thin Film

**Figure 1:**
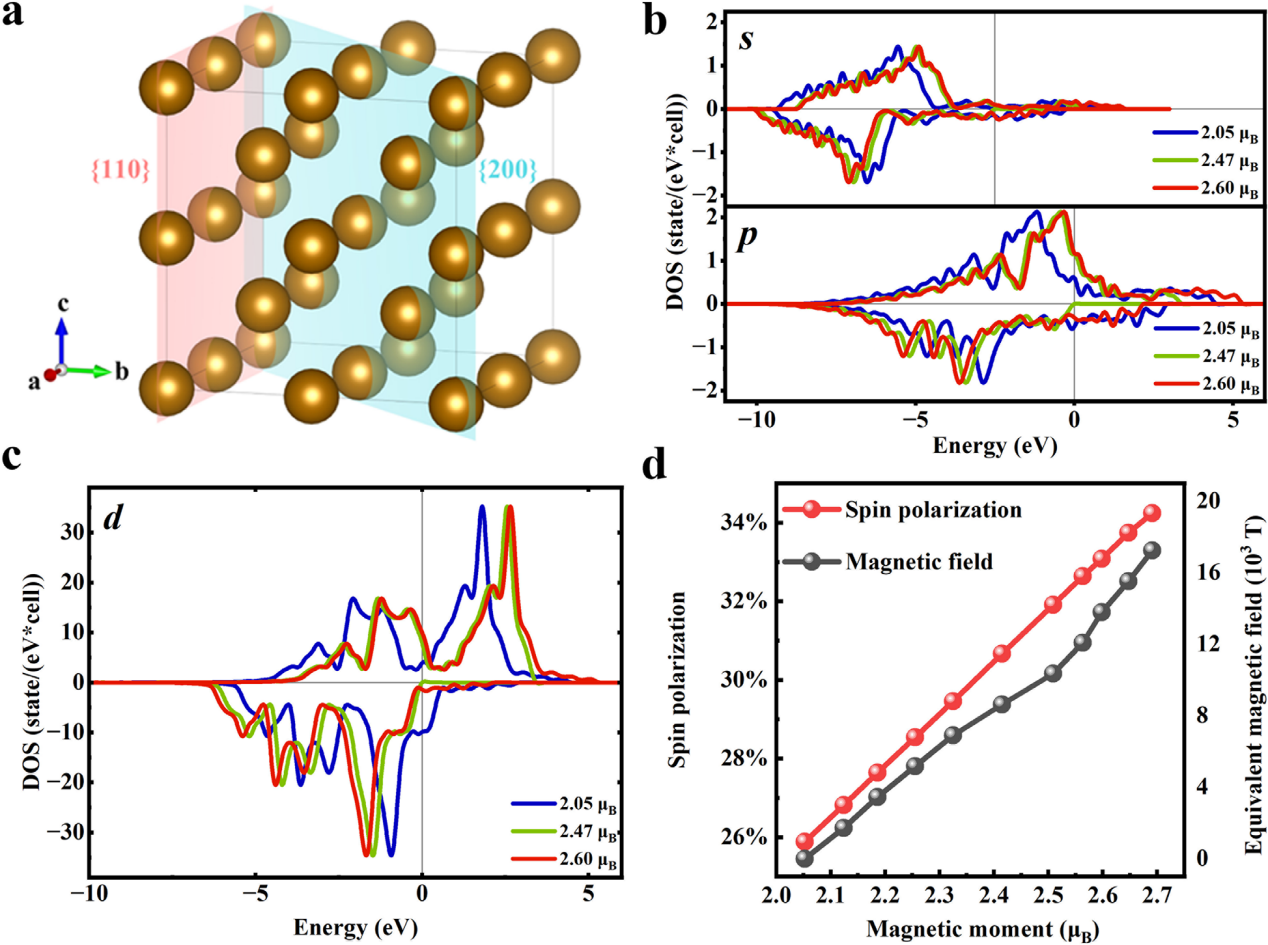
Influence of HMF on the orbital coupling of Fe. a) The calculation model of *bcc*‐Fe thin film, where the {110} and {200} planes are illustrated in the model. The DOS of b) Fe‐*d*, c) Fe‐*s*, and Fe‐*p* orbitals with the different magnetic moments of Fe atoms. The equivalent Zeeman magnetic field of 0 T, 11539 T, and 13798 T correspond to the magnetic moments of 2.05, 2.47, and 2.60 μ_B_, respectively. d) Spin polarization and applied equivalent Zeeman magnetic field as functions of the Fe magnetic moments.

To predict the modulation of growth HMF on the orbital coupling, crystallization, and magnetic properties of Fe, the first‐principles calculations were conducted to analyze the density of states (DOS) of the *s‐p‐d* orbitals in a Fe thin film. The previous research reported that the FeNi thin film grown under HMF had a higher magnetic moment.^[^ ^12^ ^]^ Since the equivalent Zeeman field in VASP simulations differs significantly from the actual magnetic field, the increase in magnetic moment was adopted to represent the enhanced growth magnetic field (see the Supporting Information for details). The field‐enhanced magnetic moments at 7 T and 9 T were estimated as 2.47 μ_B_ and 2.60 μ_B_ in our calculations, respectively. The partial DOS of the Fe thin film under different magnetic moments are depicted in **Figure** **1**b,c, respectively. As evident from Figure 1b, the DOS near the Fermi level was predominantly contributed by Fe*‐d* orbitals. Notably, the DOS of *d*‐electrons with spin‐up components exhibited a systematic shift toward higher energies, and that with spin‐down components shifted toward lower energies. This indicates that an augmentation in the Fe magnetic moment intensifies the polarization of its *d*‐electrons which directly contributes to the magnetic characteristics. Furthermore, due to the strong hybridization of *s*/*p* with *d*‐orbital, the modulation of the *d*‐electrons would inevitably influence the *s*/*p*‐orbitals. As shown in Figure 1c, the DOS of *s*‐ and *p*‐electrons had the same variation trend as *d*‐electrons. It reveals that the Zeeman energy for an increasing magnetic field could not only align the *d*‐orbital but also modulate the bonding and spin polarization of *s*/*p* valence electrons. The total spin polarization (*P*) and the equivalent Zeeman field all showed linear variation with the magnetic moment (Figure 1d). In this study, the application of a growth magnetic field was predicted to enhance the inherent spin polarization of Fe films by coupling with their atomic orbitals. The orbital arrangement was expected to be maintained even after removing the growth magnetic field since the *s*/*p*‐orbitals could freeze the spin distribution of the *d*‐orbital as well. This had profound implications for the macroscopic properties of crystal bonding and the crystallization process of the grown Fe film. Consequently, growth HMF provided a novel approach for fine‐tuning atomic orbitals, paving the way for the development of spin‐polarized materials with tailored properties.

### 2.2 HMF Influence on the Crystal Structure

**Figure 2:**
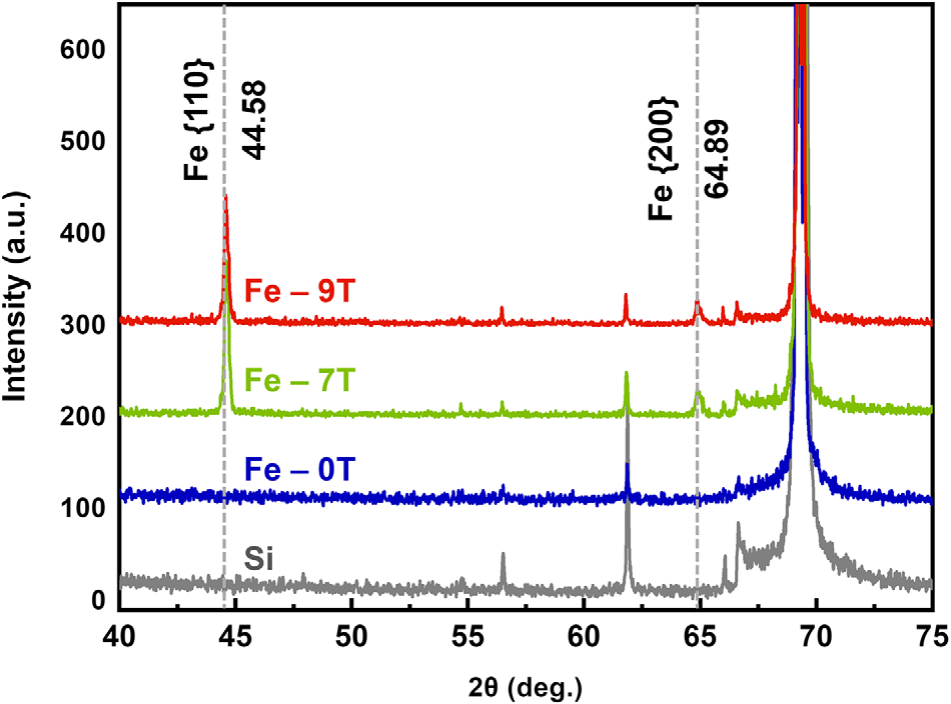
GIXRD patterns of Si (100) substrate, Fe‐0T, Fe‐7T, and Fe‐9T samples.

Theoretical results predicted that HMF could intervene in the crystal formation by regulating the orbital coupling and spin polarization of the Fe atoms. Accordingly, Fe films were grown on Si (100) substrates under magnetic fields of 0, 7, and 9 T, respectively. The substrates were all at room temperature to prevent atomic interdiffusion and enhance the manipulation of magnetic fields during the growth. Subsequently, Pd capping layers were deposited in the absence of a magnetic field. The three as‐grown samples were designated as “Fe‐0T”, “Fe‐7T”, and “Fe‐9T” for ease of identification. Considering the small thickness of the grown layers, GIXRD was employed to measure the crystal parameters of the three samples, with the comparison of the Si substrate (**Figure** **2**). The Pd capping layer well protected the Fe film from oxidation, as demonstrated by the absence of the Fe oxide peaks in the GIXRD patterns of all samples. For the Fe‐0T sample, only diffraction peaks from the single‐crystal Si substrate were observed, indicating that an amorphous structure of the Fe thin film was grown under 0 T. This may be due to the amorphous SiO_2_ layer on the Si surface, which lacked the periodic lattice information for the growth of Fe thin film. Further owing to the room temperature growth, the migration speed of deposited Fe atoms was extremely low to diffuse to the energetically favorable locations. Compared with the Fe‐0T sample, diffraction peaks located at 44.6° and 64.9° were observed both in Fe‐7T and Fe‐9T samples, corresponding to the {110} and {200} planes of polycrystalline body‐centered cubic (*bcc*) Fe, respectively. This meant the thin films were composed of a pure *α*‐phase Fe. The appearance of crystalline Fe films indicated that the HMF aligned Fe atoms by interacting with their orbitals and magnetic moments rather than driving the surface migration of Fe atoms. The magnetic interaction endowed deposited Fe atoms with ordered arrangements to form a periodic crystalline phase.

Based on the diffraction peak positions of {110} or {200} planes, the plane distances could be calculated from the Bragg formula, and the lattice constant *a* of the Fe thin films on Fe‐7T and Fe‐9T samples was further derived to be 0.2872 nm. The slightly larger lattice constant than that of Fe bulk (*a*_bulk_ = 0.2863 nm) indicates a 0.31% tension strain.^[^ ^17^ ^]^ The tensile strain might arise from the lattice deformation within the small Fe grains and might also induced by the lattice mismatch between Fe and substrate. Hence, the average grain size could be calculated according to the Debye–Scherrer formula: *D*_hkl_ = **K** *λ*/(*B*_hkl_cos**θ**),^[^ ^18^ ^]^ wherein **K** is the crystal shape factor, *λ* is the wavelength of the X‐ray, *B*_hkl_ is the full width at half‐maximum of the diffraction peak, **θ** is the Bragg diffraction angle, and *hkl* is the Miller index for the diffraction surface. The average grain size *D*_110_ of the {110} plane was calculated to be about 40 nm for Fe‐7T and Fe‐9T samples, while the *D*_200_ of the {200} plane in the Fe‐9T sample was approximately 50 nm and greater than that of the Fe‐7T sample (33.9 nm). These values were significantly greater than the Fe thickness, indicating that the Fe film was grown in two dimensions and arranged orderly by the Zeeman energy of the HMF during the growth.

### 2.3 HMF Influence on the Microstructure

**Figure 3:**
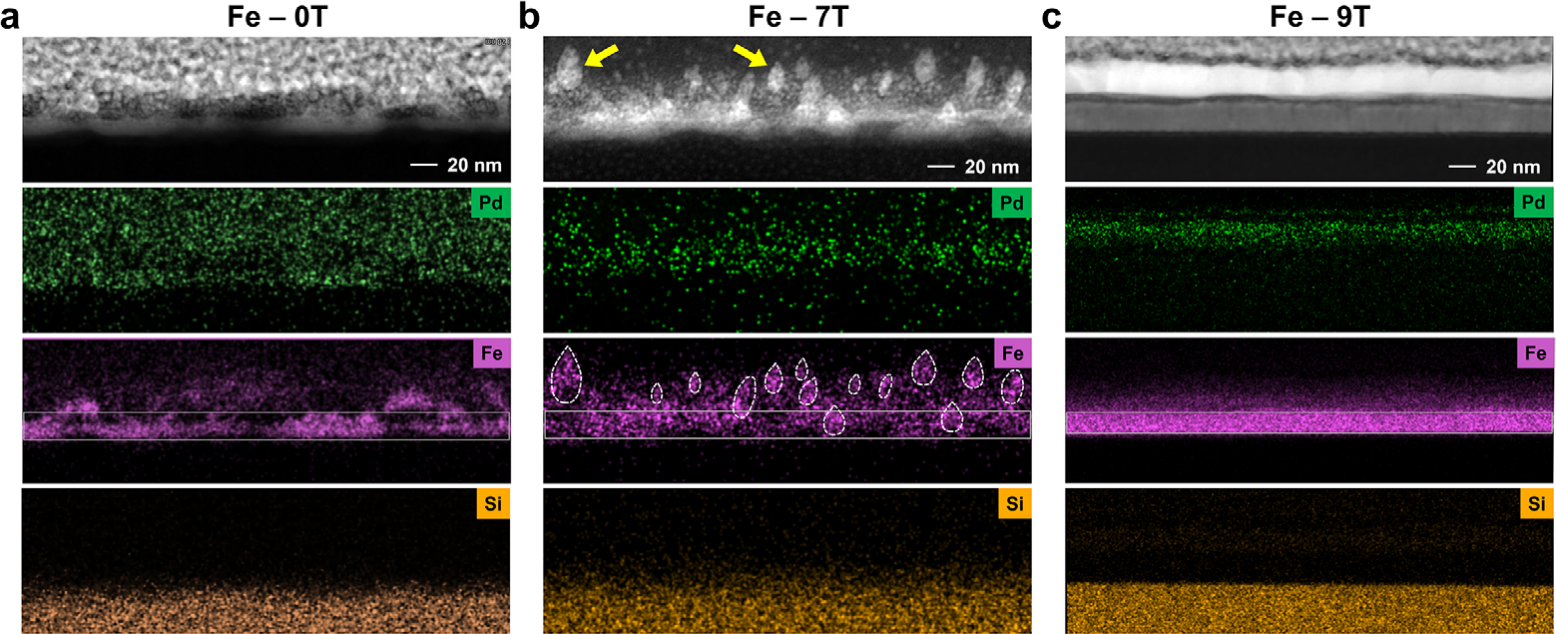
The cross‐sectional image of HAADF‐STEM and corresponding EDS analysis of the a) Fe‐0T, b) Fe‐7T, and c) Fe‐9T samples. The gray box in the figure highlights the region with the highest density of the Fe film, corresponding to thicknesses of 20, 20, and 15 nm for Fe‐0T, Fe‐7T, and Fe‐9T, respectively.

The high‐angle annular dark field scanning transmission electron microscope (HAADF‐STEM), combined with the EDS was used to detect the cross‐sectional distribution of chemical elements of the samples, as shown in **Figure** **3**. For the Fe‐0T sample, the distributions of Fe and Pd films were scattered and not uniform (Figure 3a). Although Fe and Pd layers were grown successively, the Fe/Pd interface was difficult to distinguish. The blurred interface might be caused by the scattered distribution and the surface fluctuation of the Fe film grown without an external magnetic field. For the Fe‐7T sample, the layered distribution of the Fe elements was presented with a thickness of about 20 nm (Figure 3b). Interestingly, many compact areas in the droplet shape were observed, which were contributed by the Fe layer as highlighted in the distribution image of the Fe elements. Under the modulation of 9 T HMF, the sample showed a sharp interface where both the Fe and Pd films were dense and compact (Figure 3c). Numerous droplet‐shaped Fe grains merged into a Fe film with an ordered lattice arrangement and a uniform thickness, leading to a more uniform growth of the subsequent Pd film.

**Figure 4:**
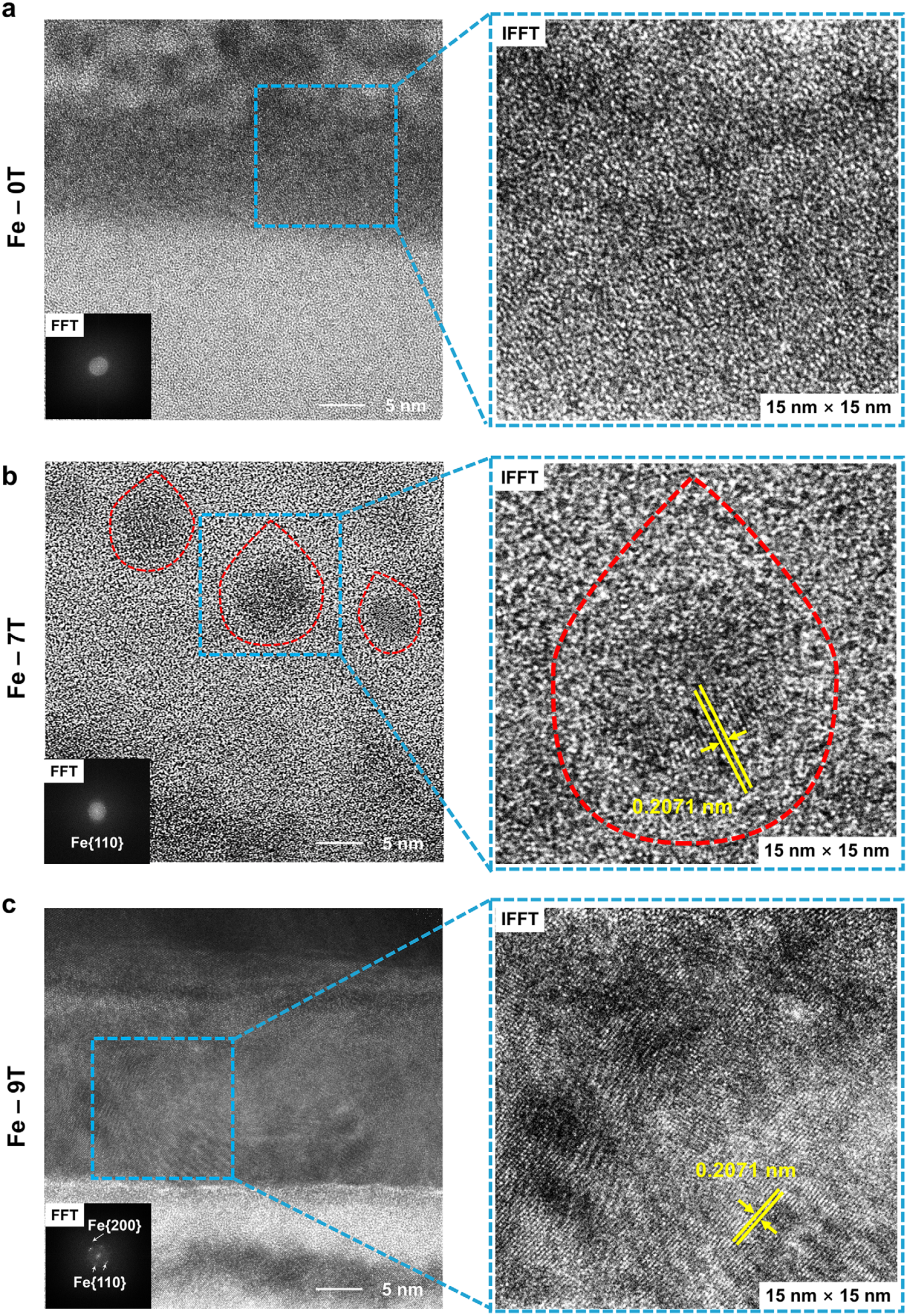
The bright‐field HRTEM images of the cross‐session structures of the a) Fe‐0T, b) Fe‐7T, and c) Fe‐9T samples. The FFT diffraction patterns and IFFT images show the crystallinity of Fe films within the blue dashed boxes. The droplet‐shaped Fe grains are highlighted by the red dashed circles.

The interface structures were further imaged through HRTEM (**Figure** **4**). No lattice fringe was observed for the Fe‐0T sample, and no obvious diffraction spot or diffraction ring could be recognized in the FFT image (in the insert of Figure 4a). This indicated the amorphous structure of the Fe film, which was further confirmed by the loose structure in the IFFT image (aligned with the GIXRD results in Figure 2). As for the Fe‐7T sample, some dense areas with darker colors could be observed by bright‐field HRTEM (Figure 4b), with the droplet shapes (corresponding to the HAADF image in Figure 3b). Different from the Fe‐0T sample, clear diffraction rings were shown in the FFT result in Figure 4b, and lattice stripes with a spacing of about 0.2071 nm were observed in the IFFT image, consistent with that of the *bcc*‐Fe {110} plane.^[^ ^19^ ^]^ This indicated that the compact distribution areas were Fe grains with *bcc* structure, and the grain growth along the magnetic field direction was significantly accelerated. The Fe‐9T sample showed a clear lattice configuration of the Fe film and the {110} plane spacing in both FFT and IFFT images (Figure 4c). The grain arrangement was tighter, with additional Fe {200} and Fe {310} diffraction patterns emerging. The above TEM characterizations of Fe/Pd films demonstrated that the growth of Fe developed from an amorphous structure to droplet‐shaped grains and 2D thin film under the increasing magnetic field.

As mentioned in the theoretical prediction, the growth mode evolution should be related to the coupling of HMF with strong Zeeman energy with the atomic orbital of Fe. Except for this, the grain shapes were further determined by the balance of demagnetization and interfacial energy.^[^ ^20^, ^21^ ^]^ From a crystallographic growth perspective, the existing Fe film and subsequently Fe grains were expected to exhibit homogeneity and complete wettability, facilitating two‐dimensional growth. Nevertheless, the demagnetization effect led to different atomic arrangements and magnetization orientations without a magnetic field, resulting in reduced density and weak magnetization. Then, numerous droplet‐shaped Fe grains emerged under the grown magnetic field of 7 T, with the aspect ratio ranging approximately from 1.69:1 to 1.75:1 (Figure 4b). This observation suggested that the change in interfacial energy per unit surface area, induced by the application of the HMF, was insufficient to solely govern the 2D crystal growth. Instead, the demagnetization energy remained a significant factor, playing a crucial role in the growth mechanism. Under the HMF, the demagnetizing field developed within the Fe grains, opposing the direction of the magnetic field. This counteracted the overall magnetic field inside the Fe grains, thus hindering the magnetic field from aligning the Fe grains with the existing Fe film and reducing their interfacial energy. Consequently, Fe grains with diverse aspect ratios were formed,^[^ ^22^ ^]^ exhibiting numerous densely packed regions of droplet‐shaped grains in the Fe‐7T sample. The dense distribution of droplet‐shaped Fe grains observed in the Fe‐7T sample suggested that the HMF has reduced the interfacial energy per unit surface area and even the total interfacial energy between Fe grains and existing Fe film, providing some allowance for the increase in demagnetization energy. Meanwhile, the magnetization increase of the Fe film brought by the HMF limited its volume expansion. Therefore, the Fe film in the Fe‐7T sample possessed a higher density than the Fe‐0T. For the Fe‐9T sample, the volume occupied by an equivalent amount of epitaxial Fe atoms was already minimal, and the total interfacial energy (*G_f_*) for the layered growth of the Fe film was minimized at *G_f_* = *σ*_mn_ *A*, where *σ*_mn_ is the interfacial energy per unit area of the germ and *A* is the surface area. In this case, any surface irregularities would increase the surface area, which aligned with the principle of minimizing free energy and favored the 2D growth of the subsequent Pd layer, the enhancement of crystalline quality, and the steepening of the interface.

It is known that the growth mode of Fe thin films depends on the contact angle between Fe atoms on the film surface and the above Fe germs, which is determined by the mechanical equilibrium conditions among the Fe thin film surface, germs, and vapor pressure.^[^ ^23^ ^]^ Assume that the Fe grain had a spherical shape, the change of Gibbs free energy of the system can be expressed as:

(1) $$\begin{array}{ccc} \Delta G & = & \left[\frac{4\pi r^{3}}{3\Omega }\left(\Delta g_{v}+C\Omega e^{2}\right)+4\pi r^{2}\sigma_{mn}\right]\left(\frac{2-3\cos\theta +\cos^{3}\theta }{4}\right)\\ & = & \left(\Delta G_{v}+VCe^{2}+A\sigma_{mn}\right)\left(\frac{2-3\cos\theta +\cos^{3}\theta }{4}\right) \end{array}$$

where *r* represents the germ radius, *Ω* is the volume of Fe atoms, *V* is the grain volume, *A* is the grain surface area, *Δg_v_* is the Gibbs free energy of Fe atoms, *e* is the elastic modulus or shear modulus of Fe crystals, and **C** is a constant related to the elastic modulus or shear modulus of Fe crystals. *Δg_v_* is always negative when Fe germs nucleated. Both the elastic energy caused by strain and the interfacial energy are always positive, which could increase the formation energy of crystal nuclei.

For the growing Fe grains, the Gibbs free energy expressed as Equation (1) must be less than zero. Therefore, Δ*G_v_* + *V* **C** *e* ^2^ + *A*σ_*mn*_ < 0, that is *V* **C** *e* ^2^ + *A*σ_*mn*_ <‐ Δ*G_v_*, where the first term, the demagnetization energy, is related to the grain volume *V*; and the second term, the interfacial energy, is related to the grain surface area A. Under the influence of an HMF, the interaction with the electronic orbitals and magnetic moments of Fe atoms (Zeeman energy) drives the atomic arrangement and magnetization orientation inside and outside the Fe grains to be consistent. For an equal amount of Fe atoms and the same crystal structure, a regular tetrahedron shape should possess the smallest volume with the least demagnetization energy. Nevertheless, a spherical shape should have the smallest surface area with the least total interfacial energy. Therefore, in the initial growth stage, the Fe grains had small volumes and large specific surface areas. The demagnetization energy played a dominant role, resulting in a spherical growth of the grains. As the grains grew, the volume and specific surface area gradually increased, the interfacial energy took place to dominate, and the grains transformed into the tetrahedron growth paradigm. As a result, grains driven by the HMF finally grew towards a conical shape along the easy magnetization direction, while maintaining a spherical shape in other directions, leading to the droplet shape observed in TEM images.

Using the elliptic model to approximate the droplet‐shaped crystal grains, the demagnetization energy can be expressed as G_d_ = 1/2 μ_0_ *V*N_∥_ *M* ^2^, thus the sum of the demagnetization energy and interfacial energy is defined as the relative free energy:^[^ ^1^ ^]^

(2) $$\begin{array}{c} G_{r}=\frac{2}{3}\;\pi r^{3}K_{s}\mu_{0}N_{∥}M^{2}+2\pi r^{2}\sigma_{mn}\left(1+\frac{{K_{s}}^{2}arcsin\sqrt{{K_{s}}^{2}-1}/K_{s}}{\sqrt{{K_{s}}^{2}-1}}\right) \end{array}$$

where μ_0_ is the magnetic permeability of vacuum, N_∥_ is the demagnetization factor, and *K*_S_ is the grain aspect ratio.

According to the minimum energy principle, the free energy is derivate concerning the grain aspect ratio *K*_S_: $\frac{\partial G_{r}}{\partial k}\;{|}_{K=K_{S0}\;}=0$, where *K*_S0_ is the optimal aspect ratio of ferrite grains under the influence of a magnetic field. By substituting the width and the aspect ratio of Fe grains extracted from HRTEM results, the demagnetization factor for a 7 T magnetic field was calculated to be 0.9272, and the relationship between the magnetization and the interfacial energy per unit area *M* ^2^ = −1.586 × 10^16^ *σ*_mn_ *A*.

It has been determined that the average Zeeman energy resulting from the application of a magnetic field significantly exceeded the average kinetic and thermal energy of the evaporated atoms.^[^ ^16^ ^]^ The average Zeeman energy increased the formation energy of Fe films, thereby exerting a notable influence on the nucleation process. Consequently, the elongation of Fe grains along the HMF direction was primarily attributed to the combined effect of magnetic dipole action and magnetic field energy. Magnetic dipoles emerged along the HMF direction,^[^ ^22^ ^]^ and attracted towards each other when the magnetic moments align with the magnetic field direction. Under the influence of the HMF, the aspect ratio of Fe grains, corresponding to the minimum relative free energy, increased. Therefore, the role of the magnetic field in the evaporation process was similar to that of other solidification and solution methods.^[^ ^1^, ^22^, ^24^, ^25^ ^]^ However, the elongation of Fe grains occurred after the initial spherical nucleus, resulting in the observed droplet grains rather than the elliptic shape. The droplet structure is more complicated and needs more exploration in the future.

### 2.4 HMF Influence on the Magnetic Properties

**Figure 5:**
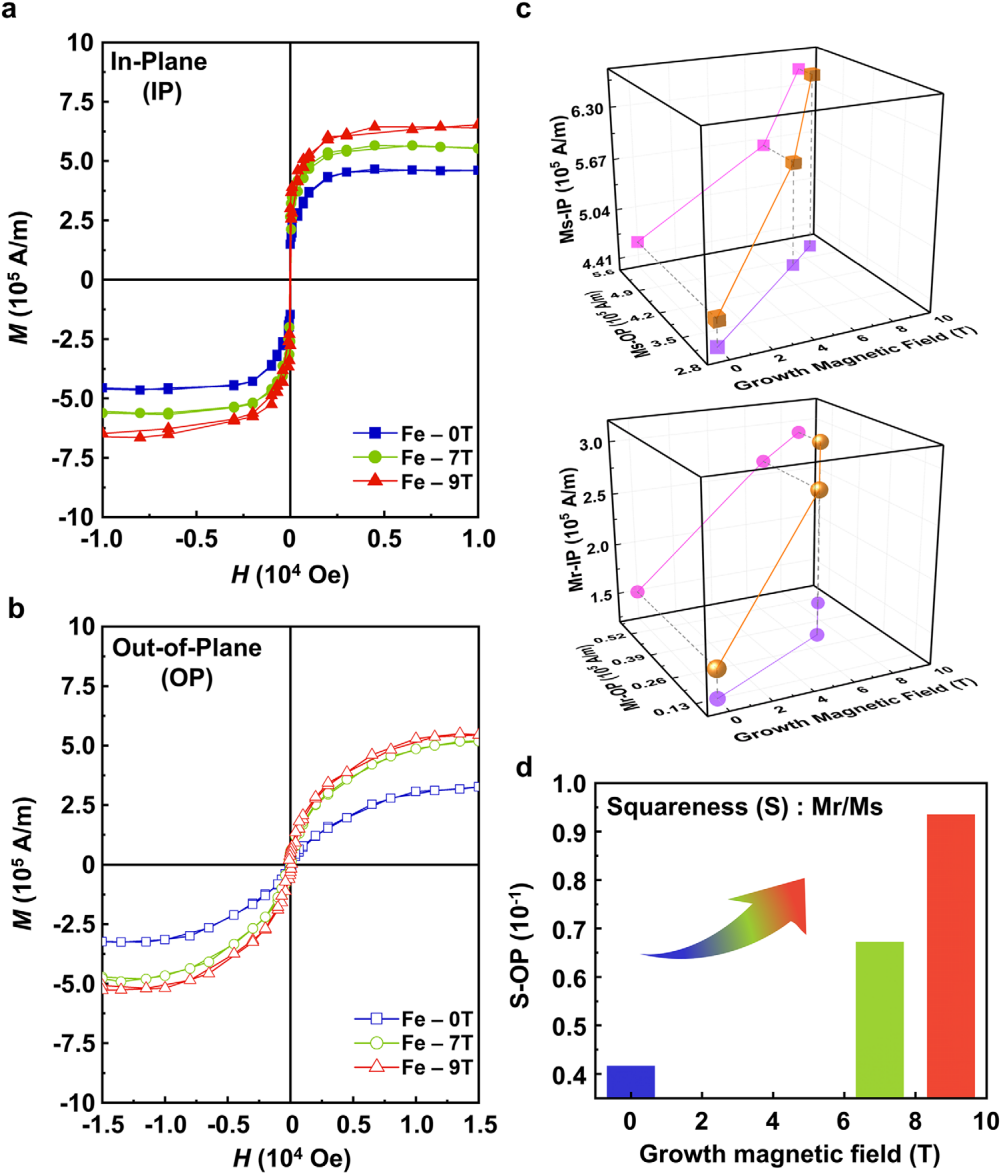
Macroscopic magnetic properties of Fe‐0T, Fe‐7T and Fe‐9T. a) IP and b) OP magnetic hysteresis loops of Fe‐0T, Fe‐7T, and Fe‐9T samples. c) *Ms* and *Mr* in IP, OP, and their vector resultant directions depend on the growth magnetic field. d) The relation between S‐OP and the growth magnetic field.

HMF played a significant role in the orbital coupling, spin polarization, and lattice arrangement, and it was expected to further modify the macro magnetic properties and micro spin texture of the as‐grown materials. To investigate the overall magnetic properties, VSM hysteresis loop measurements at angles of 0°, 30°, 60°, and 90° relative to the film plane were performed for Fe‐0T, Fe‐7T, and Fe‐9T samples, respectively (see Extended Figure 2 in Supporting Information). In‐plane (IP) and out‐of‐plane (OP) hysteresis loops were shown in **Figure** **5**a,b. In the IP hysteresis loops, the magnetic moment reached saturation at a relatively low magnetic field of approximately 0.25 T. In contrast, the OP hysteresis loops required a magnetic field of 1.5 T to reach saturation. The saturation magnetization (Ms) and the residual magnetization (Mr) in the planes of IP and OP, and the vector resultant directions of IP and OP are illustrated in Figure 5c. Compared with the saturation magnetization Ms in the Fe‐0T sample, the corresponding Ms in the Fe‐7T and Fe‐9T samples increased sequentially. The IP‐Ms in the Fe‐9T sample (6.50 × 10^5^ A·m^−1^) was enhanced by 41.9% compared with that of the Fe‐0T sample (4.58 × 10^5^ A·m^−1^). The corresponding enhancement in OP‐Ms reached 67.9%, suggesting that the crystallization of the multilayer becomes denser when raising the growth magnetic field, leading to an increase in the net spin electron per unit volume of the material. Furthermore, the Mr of the Fe‐7T and Fe‐9T samples were also enhanced by the growth magnetic field. In particular, the OP‐Mr along the growth direction was improved more significantly, showing a monotonic uptrend. Consequently, the OP squareness ratio (**S** = *Mr/Ms*) was enlarged with the growing magnetic field (Figure 5d). Evidently, the HMF had a significant impact on the saturation magnetization, residual magnetization, and squareness ratio of the Fe film, especially on its OP magnetic component.

**Figure 6:**
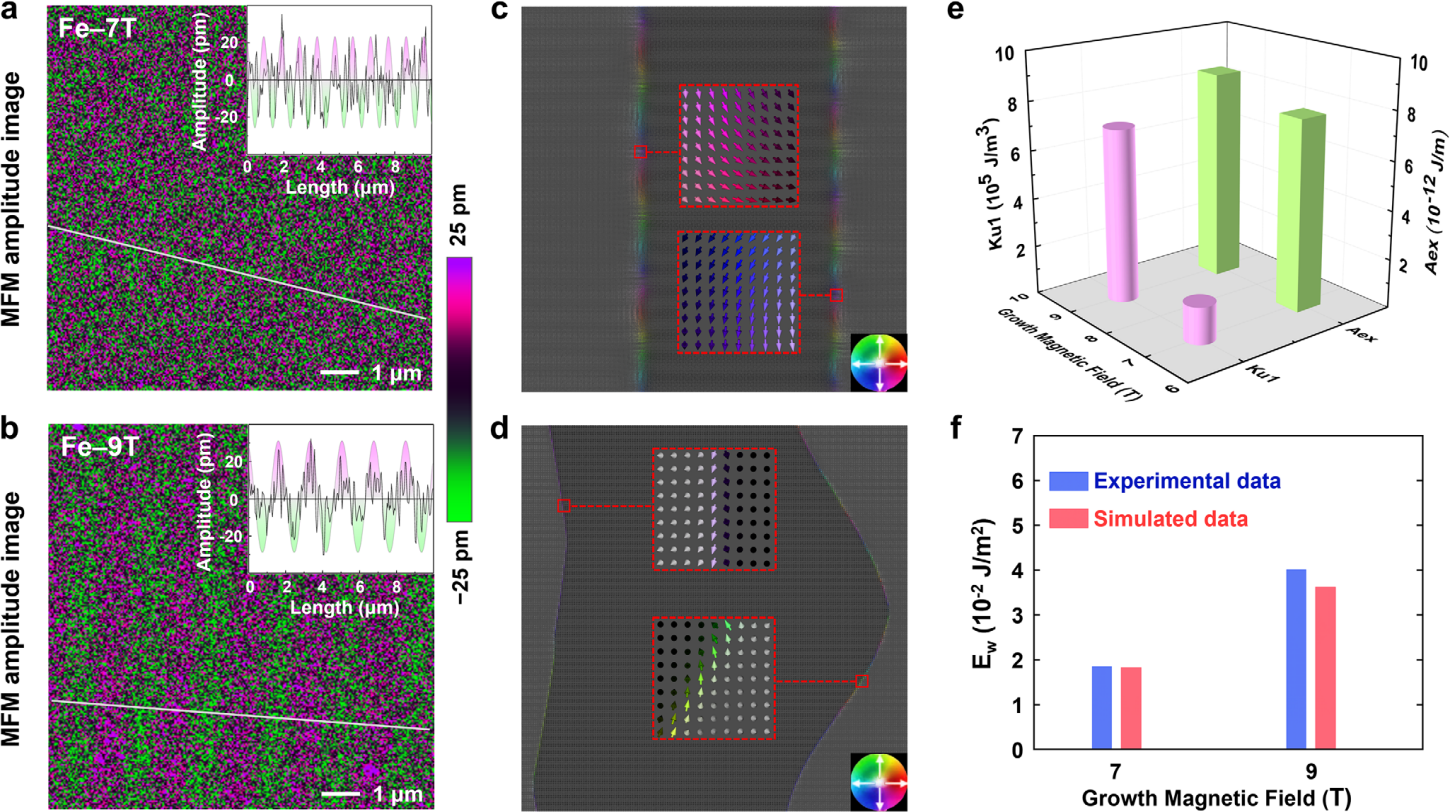
Microscopic magnetic properties of Fe‐7T and Fe‐9T. MFM images of a) Fe‐7T and b) Fe‐9T samples. The illustrations in the upper right corner are the corresponding amplitudes of white lines in (a) and (b), respectively. The simulated magnetic domain structures of the c) Fe‐7T sample and d) Fe‐9T sample. The spin textures in some areas of the domain wall are zoomed in and shown in the insert. e) The *K_u1_* and *A_ex_* of the simulated Fe‐7T and Fe‐9T samples. f) The relationship between the experimental magnetic domain wall energy and simulated magnetic domain wall energy of Fe‐7T and Fe‐9T samples.

Except for the macroscopic magnetic properties, this study simultaneously employed MFM to probe the microscopic magnetic domains of the Fe‐7T and Fe‐9T samples at room temperature. The MFM amplitude of the Fe‐7T sample exhibited alternating stripes, as shown in **Figure** **6**a. In contrast, the magnetic domains of the Fe‐9T sample exhibited more pronounced and regular periodic alternating stripes with a stronger contrast, as shown in Figure 6b. By measuring the periodic stripe domains, the average domain width could be calculated from: $D=2\sum_{j}l_{j}/\pi \sum_{j}n_{j}$, where *l*_j_ is the length of the arbitrary test line *j* intersecting the domain wall, *n_j_* is the intersection number between the test line and the domain wall.^[^ ^26^ ^]^ The changes in domain width and amplitude were shown in the insets. By fitting the cross‐section of the amplitude along the white lines in Figure 6a,b, we obtained a larger average domain width of 0.86 µm for the Fe‐9T sample than 0.48 µm for the Fe‐7T sample.

The fundamental principle for the formation of magnetic domains is to minimize the demagnetization energy.^[^ ^27^ ^]^ Magnetic thin films could reduce their demagnetizing field and demagnetization energy by forming opposite magnetization directions in adjacent magnetic domains. Consequently, the demagnetization energy density decreases with the increasing domain number,^[^ ^28^ ^]^ and researches the minimum when an equilibrium density is reached. Given the surface demagnetization energy density of $F_{d}=\frac{\mu_{0}}{2}\;{M_{s}}^{2}$ and the demagnetization energy *E_d_* = *F_d_* *L* (*L* is the film thickness), the demagnetization energies of the Fe‐7T and Fe‐9T samples were calculated to be 2.84 × 10^−3^ and 2.60 × 10^−3^ J m^−2^, respectively, by substituting the experimental results from VSM and TEM into the above formulas. The larger demagnetization energy in Fe‐7T required a greater number of domains to reduce the energy. However, the number of magnetic domains should be determined by the balance between the demagnetization energy *E*_d_ and the domain wall energy *E*_w_. The domain wall energies of the Fe‐7T and Fe‐9T samples were calculated to be approximately 1.85 × 10^−2^ and 4.01 × 10^−2^ J m^−2^, respectively, according to the relationship between the average domain width $D_{i}=\frac{10^{4}}{M_{s}}\;\sqrt{\frac{r_{w}L}{17}}$ and the domain wall energy $E_{w}=r_{w}\;\frac{L}{D_{i}}$ (where *r*_w_ is the domain wall energy density). The smaller demagnetization energy and larger domain wall energy in Fe‐9T are consistent with its fewer magnetic domains and larger domain width. Additionally, the average amplitude of the MFM signal for the Fe‐9T sample was 22.11% larger than that of the Fe‐7T sample. This explains the larger vertical magnetic moment and thus the increased OP‐Ms (Figure 5) in the film grown under a higher magnetic field.

Furthermore, micromagnetic simulations were conducted by introducing the *Ms* values of the Fe‐7T and Fe‐9T samples and adjusting parameters such as *K_u1_* and *A_ex_* to generate stripe domains. The results (Figure 6c,d) resembled those observed in the corresponding samples (Figure 6a,b). Notably, the adjacent magnetic domains possess reverse spin textures with the predominant OP magnetic moment, indicating improved magnetic structures for both cases. The two insets in Figure 6c illustrated the spin texture in typical areas of the magnetic domain wall for the Fe‐7T sample. Bloch‐type spin textures were observed distributing in a relatively wide area, indicating a gentle switching of the magnetic moments across the domain wall. This could be attributed to the low energy barrier of the magnetic domain walls or the uneven distribution of domain wall energy caused by impurities or lattice defects. Meanwhile, the spin textures rotated 180° periodically along the domain wall and were dominated by the IP components, which explained the unclear boundaries between magnetic domains in Figure 6a. In contrast, the inset in Figure 6d displayed clear Bloch domain wall within a narrow strip, exhibiting a rapid switching of the magnetic moments across the domain wall in the Fe‐9T sample. Besides, the spin textures along the domain wall were more consistent and uniform than the Fe‐7T sample. This was also aligned with the distinct domains in Figure 6b and demonstrated the role of the HMF in manipulating the crystalline quality and microscopic spin distribution. Additionally, both *K_u1_* and *A_ex_* in the Fe‐9T sample were higher than in the Fe‐7T (Figure 6e). The simulated domain wall energy of Fe‐9T was approximately 1.98 times higher than that of the Fe‐7T sample, which was close to the ratio of 2.17 times in experiments (Figure 6f, see the detailed calculation process in Supporting Information). Overall, our simulations of the magnetic domains in these two samples aligned well with the experimental data, supporting the conclusion that z‐axis anisotropy and exchange coupling positively correlate with the growth magnetic field. The results above revealed the modulation mechanism of in situ HMF on the growth of ferromagnetic materials: the HMF could determine the macroscopic magnetic properties, magnetic domain distribution, as well as spin textures of the materials by regulating the orbital coupling and spin polarization of their electronic states.

## 3 Conclusion 

In summary, first‐principles calculations were used to predict an enhanced orbital coupling and spin polarization of the Fe film when the film growth was modulated by magnetic fields. For the Fe/Pd film with Fe layer grown under increasing magnetic fields of 0, 7, and 9 T, TEM characterizations demonstrated an enhanced crystallinity of both Fe and Pd films, and the structure evolution from amorphous to crystalline was demonstrated. The presence of droplet‐shaped Fe grains under the 7 T growth magnetic field was primarily attributed to the interaction between magnetic dipole action and magnetic field energy. The augmented formation energy stemming from the competition of demagnetization energy under the 9 T growth magnetic field resulted in a more distinct interface and improvement in the crystalline structure of the Fe film. Moreover, the ameliorative magnetic properties (including *Ms*, *Mr*, magnetic domains, and spin textures) suggest a positive correlation of the z‐axis anisotropy and exchange coupling with the growth magnetic field. The HMF‐assisted MBE emerges as an effective method for regulating the crystalline structures, magnetic properties, and interfaces of FM/M heterostructures, holding promise for applications in the fields of magnetism and spintronics.

## 4 Experimental and Calculational Details

An MBE setup with a tube bore of 100 mm in diameter was installed within a cryogen‐free superconducting magnet (Cryomagnetic Model‐Custom 9 Tesla). The magnet could produce a magnetic field with the center intensity ranging from 0 to 9 T. Native oxide Si (100) substrates were placed at the position of maximum magnetic intensity. The substrate growth temperature was maintained at room temperature to further exclude the potential influence of elevated temperature on the enhancement of crystal quality. The pressure background was 1.0 × 10^−5^ Pa and remained at 8.0 × 10^−5^ Pa during the growth of Fe/Pd thin films. To refine the growth rate, the evaporation temperature of the Fe source was set at 1200 °C.

Surface morphologies were studied using atomic force microscopy (AFM, SPA400‐Nanonavi) with a scanning area of 2 × 2 µm^2^. The crystalline structures of as‐grown Fe films were measured by grazing incidence X‐ray diffraction (GIXRD, Rigaku IV) system with Cu‐Kα radiation (wavelength *λ* = 1.5405 Å) and 1° grazing angle. The chemical distribution and microstructure morphology of samples were observed by transmission electron microscopy (TEM, Talos F200X) equipped with energy dispersive spectrometer (EDS) analysis. Magnetic hysteresis loops were detected at room temperature by a vibrating sample magnetometer (VSM, MicroSense EZ9) equipped with a 360° rotational stage and having a sensitivity of 5 ×10^−7^ emu. Magnetic domain images were performed via magnetic force microscopy (MFM) using a high‐performance cobalt‐chromium reflective coating probe in a scanning probe microscope (SPM, Bruker Dimension Icon).

The Kohn–Sham density functional theory DFT using Vienna ab initio simulation package (VASP)^[^ ^29^ ^]^ was applied to study the effect of the magnetic field on Fe thin film. Generalized gradient approximation (GGA) parameterized by Perdew–Burke–Ernzerhof (PBE)^[^ ^30^ ^]^ was utilized to describe the exchange‐correlation function. Fe thin film was constructed based on a 2 × 2 × 2 supercell model of body‐centered cubic (bcc) Fe, as shown in Figure 1a. After carefully balancing computational accuracy and efficiency, the Kohn–Sham single‐particle wavefunctions were expanded in a plane wave basis set with a kinetic energy cutoff of 350 eV, while the Brillouin zone was sampled using an 8 × 8 × 8 Monkhorst‐Pack k‐point grid. To further optimize computational resource utilization, spin‐polarized calculations were performed without including spin‐orbit coupling (SOC), as preliminary tests revealed negligible differences in the DOS between SOC‐inclusive and SOC‐free calculations. Convergence criteria for energy and forces per atom were ultimately set to less than 10^−6^ eV and 0.001 eV Å^−1^, respectively. An external magnetic field was activated by the tag “BEXT”, which could set an external magnetic field that acted on the electrons. The electrons redistributed and the density changes restarted under the influence of an external magnetic field when set BEXT ≠ 0 after the charge density converged with BEXT = 0.

Micromagnetic simulations were performed in a square film of 1.024 µm × 1.024 µm using the GPU‐accelerated Mumax3 software^[^ ^31^ ^]^ by RK23 solver. The number of repetitions in *x* (*y*, *z*) to create periodic boundary conditions was 5 (5, 1), and the uniaxial anisotropy direction was set along the z‐ axis to make the simulated MFM images similar to the experimental ones (see Extended Figure 1 in Supporting Information). The uniaxial anisotropy constant (*K_u1‐7_* = 1.5 × 10^5^ J·m^−3^ and *K_u1‐9_* = 7.12 × 10^5^ J m^−3^), saturation magnetization of OP (*M_s‐7‐OP_* = 4.76 × 10^5^ A m^−1^ and *M_s‐9‐OP_* = 5.24 × 10^5^ A m^−1^), and the exchange stiffness (*A_ex‐7_* = 7.8 × 10^−12^ J·m^−1^ and *A_ex‐9_* = 8.6 × 10^−12^·m^−1^) were used for the simulations of Fe‐7 T and Fe‐9 T samples, respectively. The magnetization was initially set to be randomly distributed and then adequately relaxed by minimizing the involved energies.

The spin polarization (*P*) can be calculated using the following formula:

(3) $$P=\frac{N_{↑}\left(E_{f}\right)-N_{↓}\left(E_{f}\right)}{N_{↑}\left(E_{f}\right)+N_{↓}\left(E_{f}\right)}$$

where *N*_↑_(*E_f_*) and *N*_↓_(*E_f_*) are the densities of states above the Fermi level for spin‐up and spin‐down electrons, respectively.

## Conflict of Interest

The authors declare no conflict of interest.

## Author Contributions

X.F.W. performed the experiments and analyzed the data; B.G. performed and analyzed the First simulation analysis; X.F.W. and L.C. contributed to the sample testing; X.F.W. and Y.P.W. wrote the manuscript; X.F.W., W.Y.K., Y.P.W., X.L., and J.Y.K. revised the manuscript; X.F.W., Y.P.W., and J.Y.K. conceived this work. All authors approved the final manuscript.

## Supporting information



Supporting Information

## Data Availability

The data that support the findings of this study are available from the corresponding author upon reasonable request.
