# Supplementary material for: Orbital Coupling and Spin Textures of Fe/Pd Thin Films Grown on Si Substrate with High Magnetic Fields
Source: Adv Sci (Weinh). 2025 Apr 26;12(23):2417810. doi: 10.1002/advs.202417810 (PMC12199326; doi:10.1002/advs.202417810)
Supplement: Supplementary file 1 — Supporting Information [file ADVS-12-2417810-s001.docx]

**Orbital coupling and spin textures of Fe/Pd thin films grown on Si substrate with high magnetic fields**

Xuefeng Wu ^a, b, c^, Bin Gong ^a^, Wenyu Kang ^a^, Li Chen ^d^, Xu Li ^a^, Yaping Wu ^a*^, Junyong Kang ^a*^

1. **The calculation process of domain wall energy**

**Domain wall energy formula**:
Using the relationship for average domain width,

$D_{i}=\frac{{10}^{4}}{M_{s}}\sqrt{\frac{r_{w}L}{17}}$,

and domain wall energy,

$E_{w}=r_{w}\frac{L}{D_{i}}$,

we derive $E_{w}=\frac{17D_{i}M_{s}^{2}}{{10}^{8}}$​​, where *r*_w_ is the domain wall energy density and *L* the thickness. Substituting experimental and simulated values for Fe-7T and Fe-9T revealed significant deviations in *E_w_*_​_.

- The micromagnetic simulations employed a cell size of **1.024 µm**, aligning simulated and experimental domain widths:
  - Fe-7T: Simulated average domain width = **0.474 µm** (vs. experimental 0.48 µm).
  - Fe-9T: Simulated average domain width = **0.776 µm** (vs. experimental 0.86 µm).
- **Out-of-plane** *M*s values were uniformly adopted in simulations.
  - Fe-7T: *M_s-7-OP_*= 4.76 × 10^5^ A·m^-1^, Fe-9T: *M_s-9-OP_*= 5.24 × 10^5^ A·m^-1^.

After substituting the corresponding values into the domain wall energy formula, the experimental and simulated domain wall energies (*E_w_*) are summarized in the table below:

| **Sample** | **Experimental *E*w (J·m^-2^)** | **Simulated *E_w_*(J·m^-2^)** |
| --- | --- | --- |
| Fe-7T | 1.85×10^-2^ | 1.83×10^-2^ |
| Fe-9T | 4.01×10^-2^ | 3.62×10^-2^ |

These modifications ensured a minimized discrepancy between the simulated and experimental domain wall energy values. The simulated domain wall energy of Fe-9T was approximately 1.98 times higher than that of the Fe-7T sample, which was close to the ratio of 2.17 times in experiments.

1. **The analysis using magnetic moments instead of growing magnetic fields**

The equivalent magnetic fields employed in VASP calculations represent Zeeman-type approximations, which fundamentally differ from experimentally applied growth magnetic fields. Our primary focus lies in elucidating the magnetic moment enhancement induced by real magnetic fields and its consequential effects on intrinsic spin characteristics. This approach aligns with the principle that magnetic moment serves as the microscopic determinant of material magnetism.

Neutron diffraction studies reveal field-dependent behavior: HoFe_2_ exhibits saturation magnetizations of 2.96 μ_B_ at 4.6 T and 2.69 μ_B_ at 1.6 T [J. Phys. Condens. Matter., 1993, 5: 4077-4090], implying a linear increase of 0.1829 μ_B_ per 3 T field increment of Fe magnetic moment. Based on our VASP-calculated zero-field Fe moment (2.05 μ_B_), we estimate field-enhanced moments of 2.47 μ_B_ at 7 T and 2.60 μ_B_ at 9 T. Critically, VASP's Zeeman energy formalism neglects crucial physical mechanisms inherent to real magnetic fields – including domain reconfiguration, magnetic anisotropy evolution, and phase transitions – thereby requiring unrealistically large equivalent Zeeman fields (0 T, 11,539 T, and 13,798 T for 2.05 μ_B_, 2.47 μ_B_, and 2.60 μ_B_, respectively) to replicate experimental moment variations.

The substantial disparity between computational Zeeman fields and experimentally accessible fields (<10 T) necessitates consideration of multi-physics mechanisms during magnetic field-assisted growth:

(i) **Lorentz Force Effects**: Magnetic fields impose Lorentz forces on moving charges, modifying diffusion kinetics and crystal growth pathways. This leads to anisotropic microstructural evolution, as evidenced by the formation of droplet-shaped Fe grains and uniform crystallization patterns in **Figure. 3**, which emerge from competing energy minimization processes under field-assisted growth.

(ii) **Spin Polarization and Magnetic Ordering**: Intense fields enhance spin polarization and may stabilize unconventional magnetic states through modified exchange interactions (e.g., RKKY coupling). These changes manifest in altered electronic structures and domain configurations, exemplified by the distinct magnetic domain patterns and size variations shown in **Figure. 6**.

We therefore argue that **magnetic moment provides a more physically meaningful descriptor** than equivalent Zeeman fields for quantifying growth magnetic field impacts. While our simulations currently lack the resolution for quantitative predictions of high-field electronic orbital modifications, they enable qualitative assessment of correlations between magnetic field-assisted growth and intrinsic spin polarization characteristics in Fe-based materials.


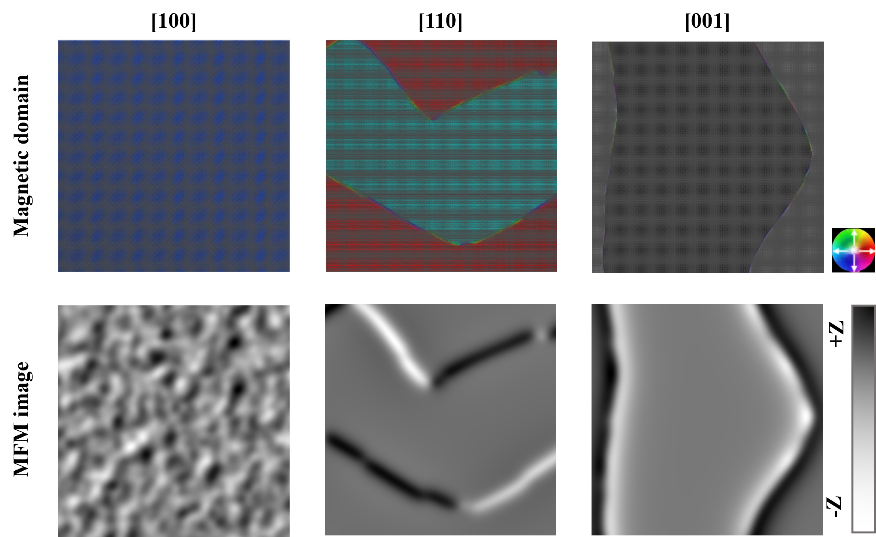


**Extended Figure 1. Simulated magnetic domain structures and corresponding MFM images obtained using Ms = 5.24 × 10⁵ A/m and Ku₁ = 7.12× 10⁵ J·m⁻³, with the uniaxial anisotropy direction oriented along the [100], [110], and [001], respectively. MFM lift height was set to 50 nm.**


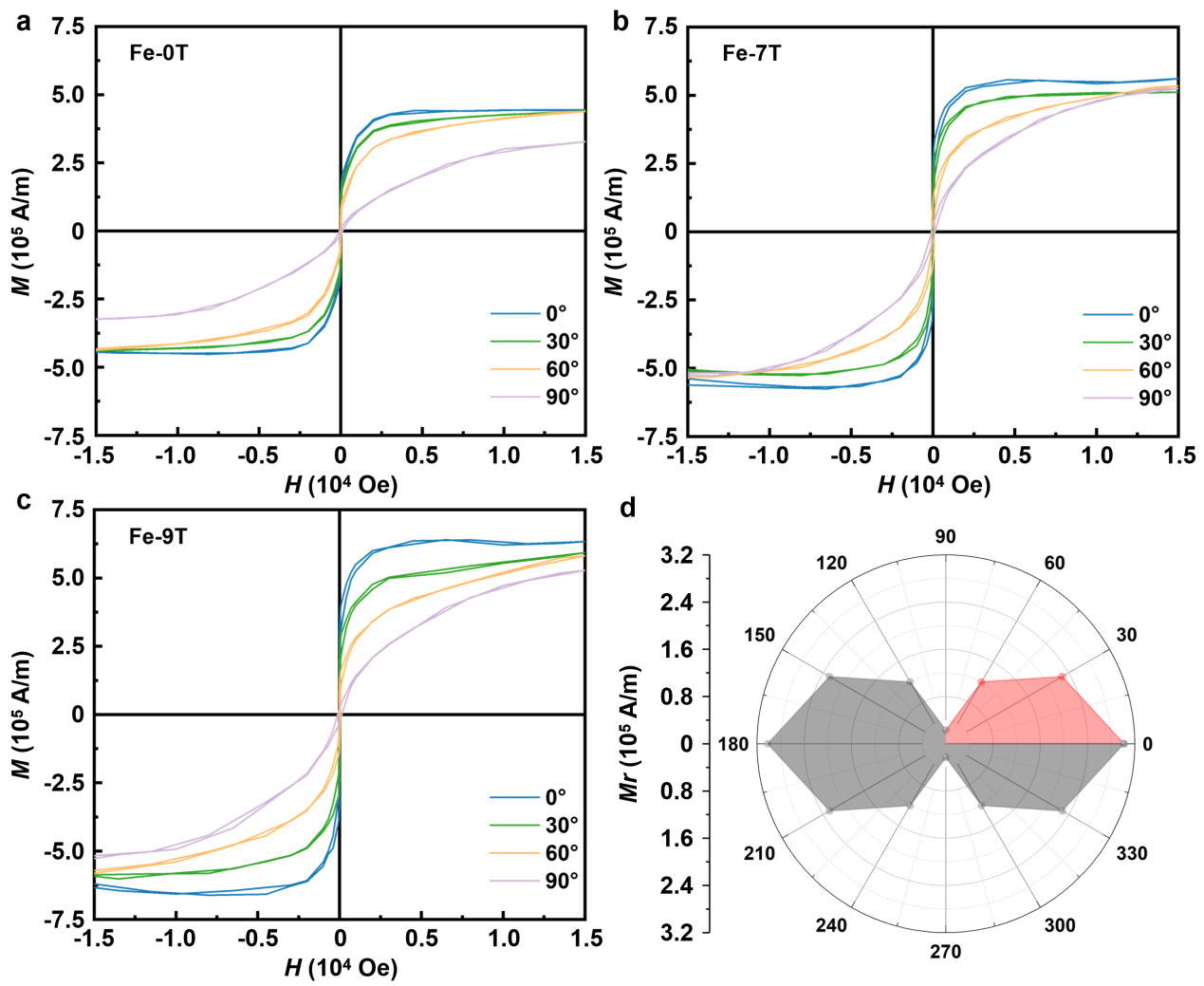


**Extended Figure 2. Hysteresis loops measured at angles of 0°, 30°, 60°, and 90° relative to the film plane of the (a) Fe-0T, (b) Fe-9T and (c) Fe-9T sample. (d) The polar plot of the corresponding remanent magnetization (*Mr*) of the Fe-9T sample. The red portion represents the actual experimental results..**
